# Sampling method influences hemolymph bacterial communities in the eastern oyster, *Crassostrea virginica*

**DOI:** 10.3389/fmicb.2026.1862013

**Published:** 2026-09-03

**Authors:** Brandon Feole, Denis Grouzdev, Emmanuelle Pales Espinosa, Guillaume Cacot, Bassem Allam

**Affiliations:** Marine Animal Disease Laboratory, School of Marine and Atmospheric Sciences, Stony Brook University, Stony Brook, NY, United States

**Keywords:** bacterial community, *Crassostrea virginica*, hemolymph, microbiome, oyster, sampling, vibrio

## Abstract

As the microbiome becomes increasingly recognized for its role in driving animal health and disease, accurate characterization of microbial community composition is essential. With this research comes the critical need for standardized sampling methods along with an understanding of the biases inherent to these methods. In oysters, hemolymph is a valuable system for microbiome research due to its diverse physiological functions, role as an indicator of health, and ease of collection. Unfortunately, much of the current literature regarding oyster hemolymph microbiome composition is opaque in its description of tissue collection methods. When these methods are clearly described, investigators often collect hemolymph via a needle inserted into the adductor muscle through a notch cut in the shell. However, due to the needle’s movement through soft tissues and mucous membranes exposed to the surrounding seawater, this notch method carries significant risk of sample contamination. An alternative hemolymph sampling method, hereafter referred to as the hole method, minimizes this potential contamination as the needle is inserted through a hole drilled in the shell directly above the adductor muscle, circumventing other soft tissues. Here, we tested whether notch and hole hemolymph collection methods produce different 16S rRNA bacterial community profiles in *C. virginica*. To compare these two sampling methods and evaluate the potential method-specific biases, particularly biases that may result from tissue contamination, oysters had hemolymph collected through both a notch and hole while alternating method order. Following taxonomic classification and differential abundance analysis of the resultant 16S rRNA sequences, the presence of disproportionate *Vibrio* spp. enrichment in notch-associated samples was evident. Notch-collected samples yielded significantly higher 16S rRNA gene copy numbers (*p* = 0.037) and a 16-fold higher relative abundance of *Vibrio* spp. compared to hole-collected samples (52.6% vs. 3.2%, p_*adj*_ = 0.006) despite no significant change in absolute counts of the dominant genus *Poseidonibacter*. Overall, this analysis demonstrates both the potential of the hole sampling method and the need for microbiome researchers to account for biases in bacterial community composition associated with their chosen sampling method.

## 1 Introduction

Along the East and Gulf Coasts of the United States, *Crassostrea virginica*, or the eastern oyster, holds immense commercial, cultural, and economic value. The eastern oyster has not only served as a persistent revenue stream through oystering and aquaculture, but also as an important food source and contributor to the structure and cultural dynamics of local communities (Keiner, 2010; National Research Council, 2004; Schulte, 2017). Furthermore, as ecosystem engineers, they play critical roles such as enhancing shoreline stability along with managing water quality through filtration and promotion of denitrification (Parker and Bricker, 2020; Scyphers et al., 2011). Despite these diverse functions, oyster populations are continually threatened by a variety of pressures including, but not limited to, disease and environmental stressors (Burreson and Ford, 2004; Beck et al., 2011; Okon et al., 2023). In response to these threats, and recognizing the unacceptable cost of losing oyster populations, multidisciplinary research efforts are underway to promote the recovery and continued success of these animals across habitats they once dominated.

One component of this research is focused on understanding the composition of oyster microbiomes. Being sessile, aquatic species that filter microorganisms from surrounding water, it is no surprise that their tissues possess complex and heterogeneous bacterial communities (Pimentel et al., 2021). However, of both greater interest and uncertainty is the functional role these community members play. Various studies have suggested potential roles in promoting ostreid herpesvirus-1 microvariant (OsHV-1 μvar) disease resistance in the Pacific oyster *Magallana gigas* and producing probiotics (Dantan et al., 2024; Desriac et al., 2014). Beyond these beneficial functions, understanding oyster microbiome composition enhances their value as sentinel species for both human pathogens and ecological disturbances. These disturbances induce distinct and characteristic shifts in oyster-associated microbial communities thus serving as an early alarm system with high relevance to human health (Walker et al., 2025).

Traditionally, research on marine invertebrate microbiomes has been focused on the gut with particularly high attention paid to those microbes thought to be associated with disease or more general dysbiosis (King et al., 2012). Increasingly though, hemolymph has risen as an attractive tissue to study various microbiome-related questions and processes. Hemolymph, the blood analog for invertebrates with open circulatory systems like oysters, was once thought to be nearly devoid of microbes (Potgieter et al., 2015; Rathour et al., 2025). This assumption was tied to hemolymph’s primary role as the driver of immune responses in these animals. Responsible for the circulation of soluble proteins involved in pathogen recognition and housing hemocytes, cells with well-studied roles in the initiation of cellular and humoral immune cascades along with the direct phagocytosing of pathogens, hemolymph was thought to simply be too hostile for any resident microbiota to flourish. However, over the past decade research has shown that hemolymph not only possesses a defined and tissue-specific microbial community, but also that this community is one of the most diverse throughout the oyster (Almuhaideb et al., 2025; Zhang et al., 2018). On top of this, and specifically due to the aforementioned immunological roles, microbiome studies in hemolymph facilitate a unique opportunity to better understand interactions between microbes and the oyster immune system (Destoumieux-Garzón et al., 2024). Finally, microbiome studies in hemolymph are advantageous as this tissue can be collected simply and efficiently, facilitates pooling of samples for higher biomass needs, and can be extracted without killing the oyster (Song et al., 2025).

Research on the role of bacteria from the genus *Vibrio* as both a causative agent of disease and predictor of disease susceptibility demonstrates the clear and pressing applications of oyster microbiome research, particularly in the hemolymph. Following the isolation of *Vibrio crassostreae* from oyster hemolymph and subsequent characterization of this species as a pathogen, numerous bacterial species from the genus *Vibrio* have been implicated as important drivers of oyster disease (Faury et al., 2004). In addition to the identification of species that contribute to vibriosis, complicated, species-level *Vibrio* dynamics have been characterized. The disappearance of *V. mediterranei*, which in other contexts has been characterized as an opportunistic pathogen, and replacement by a cascade of other *Vibrio* species marks the onset of mortality in farmed eastern oysters (Fan et al., 2023; Smith et al., 2025). Clearly, identifying members of the oyster-associated bacterial communities, especially those within the *Vibrio* genus, and understanding how their presence drives alterations in individual and population health is of significant ecological and commercial value. Moreover, given the disease states triggered by *Vibrio* presence in hemolymph, focus must be placed on accurate characterization of the oyster hemolymph bacterial community.

However, there is a critical methodological vulnerability shared among many studies investigating oyster hemolymph microbiota including the presence of *Vibrio*. A frequently used method for hemolymph collection, hereafter referred to as the notch method, consists of incising a notch in the oyster’s shell followed by insertion of a needle through the notch into the adductor muscle where the hemolymph resides (Garnier et al., 2007; Paillard et al., 1996; Wegner et al., 2025). While this method may be straightforward and common practice, it poses a significant risk of contaminating hemolymph microbiome samples. Before reaching hemolymph sinuses within the adductor muscle, the needle inevitably passes through mucous membranes covering the surface of soft tissues potentially collecting trace contaminants. Thus, due to contacting these surfaces, the needle can introduce such transient or otherwise exogenous microbes into hemolymph samples: an especially relevant risk when collecting low-microbial-biomass samples where contamination can disproportionately influence 16S rRNA community profiles. Concerningly, many studies are frequently ambiguous about, or completely omit, their sampling technique (Desriac et al., 2014; Dupont et al., 2020; Lokmer et al., 2016; Lokmer and Mathias Wegner, 2015; Zhang et al., 2018; Li et al., 2022). This creates considerable challenges in interpreting results as it becomes impossible to evaluate the potential role of contamination via non-hemolymph tissue. Such a concern is not purely theoretical. Many studies on the role of *Vibrio* bacteria or OsHV-1 in driving disease, dysbiosis, or changes in microbial community structure do not clearly describe how hemolymph samples were collected (Dupont et al., 2020; Lokmer and Mathias Wegner, 2015). Authors of the foundational research in which *V. crassostreae* was identified as an oyster pathogen and isolated from hemolymph, for instance, do not discuss how their samples were collected (Faury et al., 2004; Gay et al., 2004).

Across a diversity of study systems, method of sample collection is consistently shown to have a strong impact on the resultant bacterial community composition. For example, a comparison between noninvasive methods, specifically cloacal swabs and fecal samples, and corresponding invasive methods, direct sampling of the tissue, in birds demonstrated significant differences in both alpha (i.e., biodiversity within one sample) and beta (i.e., biodiversity between samples) diversity metrics (Zhou et al., 2023). The investigators concluded that choice of sampling method is a critical one within microbiome research and should be made after careful consideration of both the method and the specific questions being asked. In a similar manner, research comparing diabetic foot ulcer sampling methods found that alpha and beta diversity indices once again varied depending on the method used (Travis et al., 2020). It is worth noting that in both cases the methods being compared differed in degree of invasiveness. These case studies demonstrate the need for researchers to both be transparent about their methodology and consider their sampling method within the context of the specific research questions being asked.

A potentially useful alternative to the notch method avoids the previously discussed contamination risk by taking an alternative path to the hemolymph. Rather than inserting the needle through a notch cut into the edge of the shell, thus forcing it to pass through pallial fluid, mucous membranes, and possibly contact other tissues (e.g., mantle, gills), this method involves drilling a hole in the right valve immediately above the adductor muscle using sterile technique. In so doing, the needle is now able to directly enter the adductor sinuses (i.e., the same sampling location in the muscle as the notch method) through a pathway that avoids the fluids and mucous membranes encountered during standard notch sample collection. While this method may increase the time associated with sample preparation, reduced contamination could prove highly valuable particularly when studying bacterial species commonly found in non-hemolymph tissues such as those in the *Vibrio* genus (King et al., 2021). Though this method has been used previously, no work has been done to evaluate the risk of contamination for either this or the notch method (Almuhaideb et al., 2025). Such an analysis is important for advancing the study of oyster hemolymph microbiomes due to the impact of sampling method on resultant microbial community composition clearly evidenced by the aforementioned case studies in other systems (Travis et al., 2020; Zhou et al., 2023). In addition to evaluating whether one method is preferable to the other, characterizing differences in bacterial community composition between two hemolymph sampling methods used in oyster microbiome studies may identify biases that researchers should control for when using them.

The study described herein seeks to address two primary hypotheses regarding the impact of sampling method on derived oyster hemolymph bacterial communities. First, we hypothesize that the specific method used to collect oyster hemolymph will distinctly shape the observed bacterial community composition affecting both overall taxonomic profiles and the differential abundance of specific taxa. Second, due to its direct access of internal muscle tissue and elimination of contact with soft tissue-associated mucous membranes, we postulate that the hole method may introduce lower levels of bacterial contamination into the hemolymph compared to the notch method. To evaluate these hypotheses, hemolymph samples were collected from oysters using both methods. DNA within these samples was then extracted followed by amplification and sequencing of 16S rRNA. A computational pipeline incorporating statistical approaches including permutational analysis of variance (PERMANOVA) and differential abundance (DA) analysis was deployed to characterize and compare bacterial community composition as a function of sampling method. Based on the results of this analysis, which demonstrate significant differences (i.e., biases) in the taxonomic composition of bacterial communities associated with hemolymph sampling methods, recommendations are made for future microbiome research in marine bivalves such as oysters.

## 2 Materials and methods

### 2.1 Oyster source and holding conditions

Experimental protocols used in this study were reviewed and approved by Stony Brook University (NY, United States) and conducted in accordance with institutional, state, national, and international guidelines governing the ethical use of animals in research. Oysters (18-month-old, approximately 7.5 cm in length) were collected in November 2024 from Napeague Harbor, East Hampton, NY, and immediately transported to the Marine Animal Disease Laboratory at Stony Brook University in Stony Brook, NY, United States. Upon reception, all oysters were immediately transferred to a 40-gallon tank operating as a closed-loop recirculating aquaculture system and held at approximately 18 °C with a salinity of 28–30 ppt. The animals were acclimated to this standard laboratory temperature to reduce variability stemming from changing environmental conditions in NY during fall, while salinity was maintained to reflect original field conditions. The tank water was changed twice a week during the holding period and was continuously filtered through a chemical filtration module (Lifegard, Model AF-93-19) containing layered granular activated carbon and porous mineral bio-media. Seawater for holding tanks was sourced from the entrance channel of Stony Brook Harbor (coordinates: 40.9211, −73.1503). Oysters were fed twice daily with shellfish diet algal paste (Reed Mariculture, Shellfish Diet 1800, Campbell, CA, United States) following manufacturer’s recommendations; feeding was withheld on the day of sampling, however, to minimize potential sample contamination and confounding metabolic artifacts. Hemolymph was sampled 49 days post oyster-collection. This extended holding period was chosen to ensure uniform acclimation to laboratory conditions, thereby mitigating the pronounced seasonal variability present at the time of oyster collection.

### 2.2 Hemolymph collection and DNA extraction

#### 2.2.1 Hole method

**FIGURE 1:**
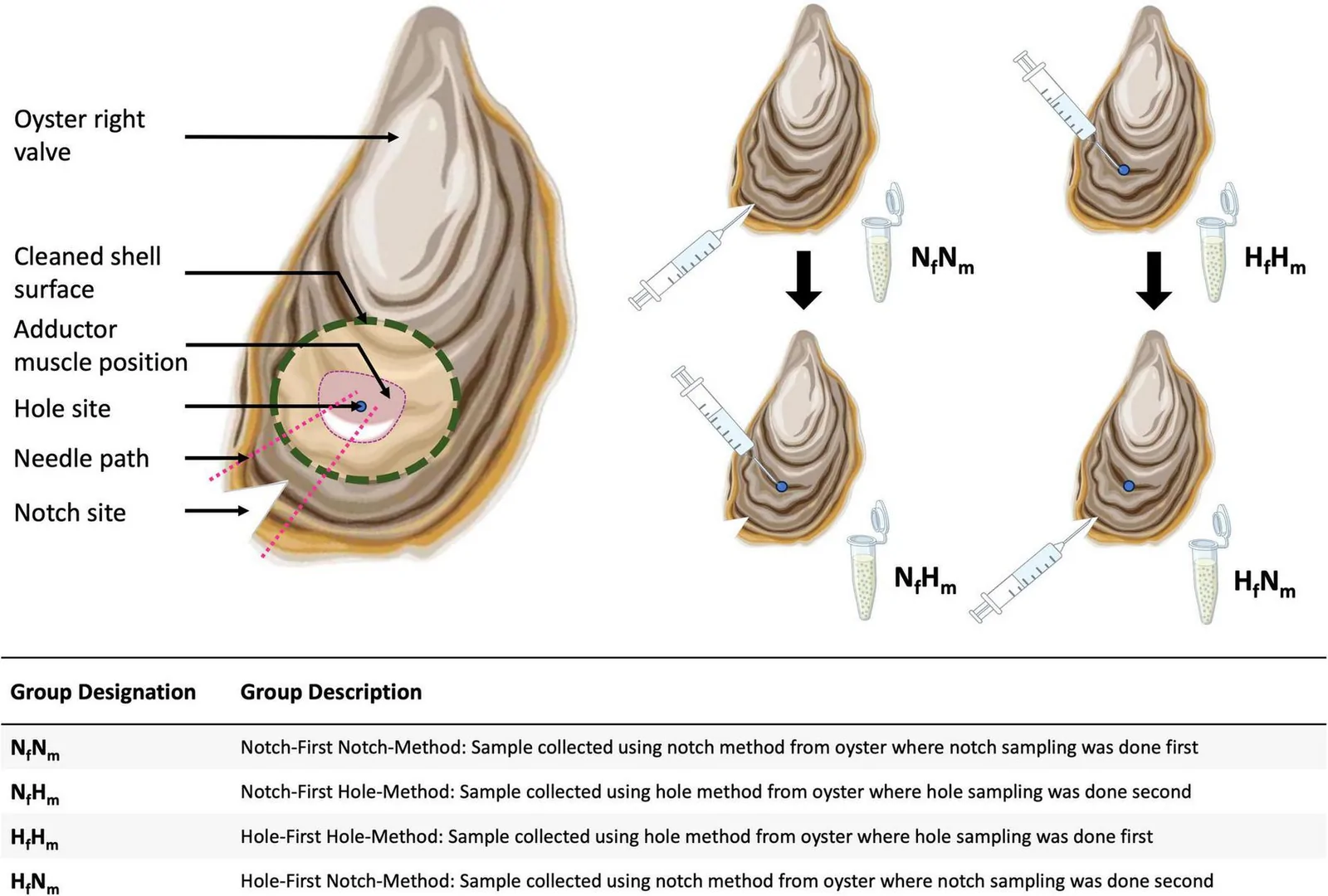
Methods diagram showing the notching and hole drilling sites along with the notch-method needle path; a visual representation and associated table entry for each sample “group” is included.

A region of the right valve directly overlying the adductor muscle was cleaned through surface abrasion using a small grinding wheel attached to a rotary tool followed by sterilization using 70% ethanol. The hole was then drilled directly above the adductor muscle (Figure 1) using a rotary tool equipped with a sterilized diamond grinding needle. This step was performed carefully to ensure that the grinding needle did not enter the adductor muscle. While drilling, sterile air, generated by attaching a 0.22 μm filter to the end of bleach-sterilized tubing connected to a diaphragm air pump, was continually blown over the hole to remove shell debris. Once the hole was drilled, an 18-gauge needle was inserted into the adductor muscle and the hemolymph was withdrawn.

#### 2.2.2 Notch method

The notch was incised on the ventral, posterior margin of the shell near the adductor attachment point (Figure 1) using a pair of heavy-duty bone shears. Following this, an 18-gauge needle was inserted into the adductor muscle (same general location in the muscle, i.e., angle and depth- as for the hole method) and hemolymph was withdrawn.

#### 2.2.3 Experimental design

Oysters were divided into two groups designated notch-first (N_*f*_) and hole-first (H_*f*_). N_*f*_ oysters had hemolymph collected first via the notch method (N_*m*_) and then via the hole method (H_*m*_) immediately afterward, whereas H_*f*_ oysters had hemolymph collected using the same methods but in opposite order. This produces the following four groups by which hemolymph samples are categorized: notch-first notch-method (N_*f*_N_*m*_), notch-first hole-method (N_*f*_H_*m*_), hole-first hole-method (H_*f*_H_*m*_), and hole-first notch-method (H_*f*_N_*m*_) (Figure 1).

A total of 20 oysters were processed in groups of five individuals at a time. Oysters within this group of five were held on ice, whereas all others remained in their respective tanks. Both sample types were taken consecutively from each oyster with all samples being collected within an approximately 6-h time frame. Oysters were sampled in the order of 5 N_*f*_, 5 H_*f*_, 5 N_*f*_, and 5 H_*f*_. For all samples, 300 μL of hemolymph was collected, transferred into 1.5 mL microcentrifuge tubes, and immediately placed on ice. Upon completion of hemolymph collection, samples were transferred to an ultra-low temperature freezer for storage at −80°C. To reduce the introduction of further background contamination or other sources of noise, no anticoagulants, stabilization buffers, or chemical preservatives were added to the samples prior to freezing.

The following environmental controls were collected: three 1 mL aliquots of seawater from the tanks in which oysters were held (tank water; TW), three 1 mL aliquots of filtered artificial seawater (FASW) collected using the same syringes and needles as used to collect the hemolymph (syringe and needle; SN), and three 1 mL aliquots of FASW were collected after being bubbled with the same filtered air used for hole method sampling (air controls; AC). The artificial seawater (ASW) used for SN and AC controls was produced by mixing distilled water with Instant Ocean Marine Fast Dissolving Sea Salt to a salinity of 28 ppt to match holding tank conditions. This ASW was then filtered through a 0.22 μm sterile syringe filter to minimize environmental bacterial contaminants. Bubbling for AC controls was done by submerging the tip of the filtered air delivery system into FASW and running the air pump before collecting aliquots of this bubbled water.

#### 2.2.4 DNA extraction

DNA extraction was done using the Qiagen DNeasy PowerSoil Pro kit following the manufacturer’s protocol. The PowerBead Pro Tubes were from Lot No. 17819977 and Mat. No. 1108822 whereas all solutions were from Lot No. 178019166. For the 40 hemolymph samples, each collected as a single biological unit, hemolymph was subdivided into 75 μL aliquots generating technical triplicates for DNA extraction. This multi-extraction framework was adopted to account for the potential limitation of DNA extraction stochasticity and ensure representative sampling of low template events (Bender et al., 2018; Erb-Downward et al., 2020). Nine sampling controls, described in the above section, were similarly extracted with each aliquot representing one technical replicate and thus not extracted in triplicate. Two additional control types, 75 μL of ultrapure water processed through the entire extraction process (extraction controls; EC) and 50 μL of unadulterated elution buffer (solution controls; SC), were also generated in triplicate. All samples except SC controls were eluted in 50 μL of elution buffer. Extracted DNA was stored at −80°C ultra-low temperature freezer. This resulted in a total of 134 extracted DNA subsamples due to the loss of one SN replicate.

### 2.3 Quantitative PCR assays

To evaluate the gene copy numbers of 16S rRNA in extracted DNA samples a quantitative PCR was conducted using primers 341F (*5′-CCTACGGGAGGCAGCAG-3′*) and 518R (*5′-ATTACCGCGGCTGCTGG-3′*) to amplify the V3 hypervariable region (Muyzer et al., 1993). To derive copy number values a gBlock sequence, designed to recreate the V3 region of the *Escherichia coli* 16S rRNA sequence (NCBI GenBank accession no. J01859), was run alongside the samples with concentrations ranging from 10^2^ to 10^8^ copies/μL (Ehresmann et al., 1972). The gBlock Gene Fragment sequence was generated by IDT based on a provided sequence (Supplementary Data 1). Each DNA extraction subsample was run in triplicate with reactions having the following composition: 5 μL Takyon No ROX SYBR MasterMix blue dTTP, 0.9 μL 50% glycerol, 0.7 μL ultrapure water, 0.2 μL of each primer at 10 μM concentration, and 3 μL of template DNA. Quantitative PCR (qPCR) was performed using a Thermo Fisher QuantStudio 6 Flex with each cycle consisting of an initial 3-min denaturation step at 98°C followed by 40 cycles of a 15-s 98°C denaturation step, a 30-s 55°C annealing step, and a 20-s 72°C extension step. For the melt curve stage, samples were held at 95°C for 15 s before being decreased to 65°C for 15 s. The temperature was then brought up to 95°C at a rate of 0.1°C /s during which fluorescence data was collected before being held for 15 s (Koziaeva et al., 2025).

A standard curve was generated using the known copy number and threshold cycle (Ct) of the gBlock standards. 16S rRNA copy number per μL of extracted DNA for each sample was then calculated using the standard curve equation and the sample’s Ct values averaged across the technical qPCR (not DNA extraction) replicates. Copy number outliers within DNA extraction technical replicates were removed using a modified Z-score threshold of 3.5 based on the median absolute deviation. To determine if 16S rRNA copy number varied as a function of “group” or “first” (Table 1), a Kruskal-Wallis rank sum test was run with copy number values as the response variable (Kruskal and Wallis, 1952). To evaluate the impact of “method,” 16S rRNA copy numbers were first *log*_10_-transformed to satisfy normality assumptions. A one-tailed paired Student’s *t*-test was performed on this dataset to compare N_*m*_ and H_*m*_ samples thus evaluating the hypothesis that notch sampling would yield higher copy numbers due to potential contamination.

**TABLE 1:** Description of sample clustering strategies, also used as predictor variables in statistical models.

Clustering strategy	Description	Compared groups
“Group”	N_*f*_N_m_, N_*f*_H_m_, H_*f*_H_m_, and H_*f*_N_m_; described in Figure 1	NA
“First”	The first sampling method used for a given oyster: notch-first versus hole-first	N_*f*_N_m_+N_*f*_H_m_ vs. H_*f*_H_m_+H_*f*_N_m_
“Method”	The method used to collect a sample: notch-method versus hole-method	N_*f*_N_m_+H_*f*_N_m_ vs. N_*f*_H_m_+H_*f*_H_m_
“Order”	The order in which a given sample was taken: first sample versus second sample	N_*f*_N_m_+H_*f*_H_m_ vs. N_*f*_H_m_+H_*f*_N_m_

### 2.4 16 S rRNA amplicon sequencing and quality control

Paired-end sequencing (2 × 250 bp) of 16S rRNA gene amplicons spanning the V3–V4 region (primers 341F/806R) was performed by Novogene (Tianjin Medical Laboratory, Tianjin, China) on an Illumina NovaSeq 6000 platform. Library preparation followed Novogene’s standard protocol with recovered DNA fragments undergoing end-repair, A-tailing, and ligation with Illumina-specific sequencing adapters. These fragments were then PCR amplified with their integrity and size evaluated using an AATI Fragment Analyzer and the effective concentration of the library measured using qPCR.

Raw paired-end reads were processed using USEARCH v11.0.667 (Edgar, 2010). Primer-derived sequence was removed by truncating reads (-fastx_truncate), followed by quality filtering to retain high-quality reads (-fastq_filter). Chimeric sequences were identified and removed using UCHIME (Edgar et al., 2011) as implemented in USEARCH. Denoising was then performed with UNOISE3 (Edgar and Flyvbjerg, 2015) to infer error-corrected sequence variants. These variants were subsequently clustered into OTUs at 98.5% sequence identity using USEARCH (-cluster_fast; -id 0.985). This threshold, though associated with some loss of species-level resolution and thus the potential merging of closely related taxa, allows for the collapsing of intragenomic 16S rRNA copy variation while retaining biologically meaningful differences between distinct taxa (Rodriguez-R et al., 2018). An OTU table was generated by mapping quality-filtered reads to OTU representative sequences (-otutab). Technical replicates were collapsed to the sample level by averaging OTU relative abundances across replicates prior to downstream analyses. To mitigate potential contamination during the experimental process, decontamination was performed using the R package “decontam” based on the sequences of the negative controls (Davis et al., 2018). To eliminate biases in diversity metrics associated with uneven sequencing depth, all samples were normalized to a uniform depth of 10,000 reads per sample. Technical replicates yielding fewer than 10,000 effective reads were excluded with all remaining libraries being uniformly rarefied to this threshold value. This threshold was chosen to ensure robust coverage of the hemolymph bacterial communities while optimizing sample retention. Alpha- (Supplementary Data 2) and beta-diversity (Supplementary Data 3 for Weighted UniFrac) metrics were calculated from the OTU table using the USEARCH -alpha_div and -beta_div commands, respectively. Representative OTU sequences were assigned taxonomy using the SILVA SINA aligner (v1.2.12) against SILVA release 138.2 with default parameters. Phylogenetic analysis was performed for OTUs identified as most informative in the DA analyses. Representative 16S rRNA gene sequences were aligned using MUSCLE (Edgar, 2004). The maximum-likelihood phylogeny was inferred in IQ-TREE (Nguyen et al., 2015) using the GTR + F + I + G4 substitution model selected by ModelFinder (Kalyaanamoorthy et al., 2017) and the branching support was estimated using UFBoot2 (Hoang et al., 2018).

#### 2.4.1 Alpha diversity analysis

To evaluate differences in univariate alpha diversity (i.e., biodiversity within one sample) metrics between sample groups, a series of four linear models (LMs) was constructed with the predictor variables set to “group,” “first,” “method,” and “order,” respectively (Table 1). For “group” and “first” models, an unpaired design was used whereas a pairwise linear mixed-effects (LME) model with OysterID set as the random effect was used for “method” and “order.” All models were run in Python (v3.14.0) with unpaired and paired LMs fit using the ols and MixedLM class from the *statsmodels* (v0.14.6) package, respectively (Seabold and Perktold, 2010). Each model was then run using both the Simpson and Shannon alpha diversity index as the response variable (Shannon, 1948; Simpson, 1949).

#### 2.4.2 Beta diversity analysis

Permutational multivariate analysis of variance (PERMANOVA) was performed using the *adonis2* function in the *vegan* package (v2.7.2) within the R environment (v4.5.1) to assess comparisons of beta diversity (i.e., biodiversity between samples; Anderson, 2001). Group dispersion was independently evaluated using the *betadisper* function in the *vegan* package (Anderson, 2001). Distances between samples, derived from the weighted UniFrac tables output by the USEARCH -beta_div command, served as the response variable for all PERMANOVA models (Lozupone et al., 2007). Four permutational models were generated for each of the described clustering strategies (Table 1). For “group” and “first” an unpaired design assuming independent observations was used. For “method” and “order” a paired model design was used with strata set to OysterID. Due to the paired nature of these models the weighted UniFrac matrix was subset to exclude samples without a corresponding pair. Comparisons between each unique pair of “group” identity were conducted using a similar approach but with the predictor variable set to group identity. For example, to compare N_*f*_N_*m*_ and H_*f*_H_*m*_, the data was subset to only those groups with H_*f*_H_*m*_ identity serving as the predictor variable. The final two comparisons were between N_*f*_N_*m*_ versus all other samples and H_*f*_H_*m*_ versus all other samples. Relationships quantitatively described by PERMANOVA models were visualized as Principal Coordinate Analysis (PCoA) plots using the *ggplot2* package (v4.0.1) run in the R environment (Wickham, 2016).

All generated *p*-values were corrected using the Benjamini-Hochberg (BH) method to control the false discovery rate (Benjamini and Hochberg, 1995). For these multiple testing corrections, hypotheses were grouped into three categories: primary hypotheses, pairwise comparisons, and planned contrasts. Primary hypotheses included those tests based on the original four clustering strategies (Table 1), pairwise comparisons were between each unique pair of “group,” and planned contrasts were the models comparing N_*f*_N_*m*_ and H_*f*_H_*m*_ versus all other samples. Multiple testing correction was applied within but not between these groups of hypotheses to avoid overcorrection across conceptually distinct hypothesis families (Benjamini and Hochberg, 1995).

#### 2.4.3 Compositional abundance analysis

Differentially abundant taxa were identified using the *ancombc2* function in the R package *ANCOMBC* (v2.12.0) (Lin and Peddada, 2024). A prevalence filter of 0.2 was applied to address structural zeroes in the data and prevent overrepresentation of rare taxa. A total of 11 comparisons were conducted, three of which using our main categorical variables of “method,” “first,” and “order.” Similar to the PERMANOVA models, DA analysis was conducted for all six unique pairwise comparisons of “group.” For comparisons based on “method” and “order,” along with pairwise “group” comparisons where “first” was shared (e.g., N_*f*_N_*m*_ versus N_*f*_H_*m*_), a random effect of OysterID was incorporated into the model and the data were subset to include only samples that had a matching pair nested within OysterID. The final two comparisons were N_*f*_N_*m*_ and H_*f*_H_*m*_ versus all other samples, respectively. BH correction was applied to all *p*-values for taxa within an individual comparison.

To focus subsequent compositional analyses, 21 OTUs across 14 families, hereafter referred to as “important OTUs,” were selected based on reaching ≥ 1% relative abundance in at least one sample combined with either detection in ≥ 2 samples or a maximum relative abundance exceeding 10% in at least one sample (Table 2). Taxonomic assignments for the OTUs were verified by BLAST searches against both NCBI GenBank and EZBioCloud type strain databases (Sayers et al., 2025; Yoon et al., 2017). A phylogenetic tree showing the relationship between these important OTUs, including separation by class taxonomy and clustering within closely related groups of taxa, is presented in Supplementary Figure 1.

**TABLE 2:** Taxonomic information, relative abundance, and prevalence of important OTUs.

			Best Hit		
OTU ID	Relative abundance (% of total reads)	Prevalence (no. of samples)	Sequence identity (%)	NCBI accession	Taxon
OTU1	1.11	20	99.1	CCJW01000022	*Vibrio crassostreae*
OTU3	0.16	15	99.1	ABCH01000080	*Vibrio shilonii*
OTU4	0.56	18	99.5	MN381951	*Maritimibacter harenae*
OTU18	0.31	17	98.8	NR_042467	*Vibrio cyclitrophicus*
OTU29	2.36	22	99.8	X74724	*Vibrio splendidus*
OTU31	0.25	15	100.0	MW132956	*Shewanella aegiceratis*
OTU50	1.08	16	90.6	HQ434766	*Phaeocystidibacter luteus*
OTU59	1.29	12	99.1	REFJ01000011	*Umboniibacter marinipuniceus*
OTU60	0.38	3	99.5	AABF01000026	*Fusobacterium vincentii*
OTU110	4.73	26	100.0	EF192392	*Shimia litoralis*
OTU127	**49.61**	29	99.0	LT629298	*Poseidonibacter lekithochrous*
OTU130	0.12	12	98.1	KJ728849	*Polaribacter lacunae*
OTU133	0.31	16	99.5	AJ316199	*Vibrio chagasii*
OTU137	0.38	2	90.6	LT716015	*Mycoplasma todarodis*
OTU153	0.26	13	96.3	AF118021	*Enterovibrio calviensis*
OTU179	**20.88**	28	100	FLLQ01000028	*Vibrio mediterranei*
OTU180	0.64	7	99.8	OR999466	*Zobellia uliginosa*
OTU203	0.56	3	99.8	RAQM01000002	*Tenacibaculum lutimaris*
OTU215	0.57	4	99.5	FM995181	Uncultured spirochete SRODG048
OTU223	0.50	15	99.5	LC582327	*Mycolicibacterium cyprinidarum*
OTU255	0.48	1	88.6	LT716015	*Mycoplasma todarodis*

Bolded relative abundance numbers indicate values associated with the two dominant OTUs in terms of compositional abundance.

To quantify differences in abundance, a Kruskal-Wallis rank sum test was performed using relative abundance and qPCR-scaled absolute abundance (representing estimated genus-specific 16S rRNA gene copy numbers) belonging to the *Vibrio* or *Poseidonibacter* genera for each sample with “group” serving as the predictor variable (Kruskal and Wallis, 1952). This qPCR-scaled absolute abundance was estimated by multiplying the relative abundance of OTUs belonging to the two genera of interest by the estimated 16S rRNA gene copy number in each sample based on qPCR. A Dunn’s *post-hoc* pairwise comparison with Holm-Bonferroni *p*-value adjustment was then conducted for each unique pair of “group” identity to determine which specific pairs differed in abundance of *Vibrio* and *Poseidonibacter* bacteria (Dunn, 1964; Holm, 1979).

## 3 Results

### 3.1 Quantitative PCR assays

An approximately 1,700-fold difference was observed in terms of 16S rRNA gene copy numbers across samples with a maximum, belonging to sample O10N_*f*_N_*m*_, of 6.2 ± 3.8 × 10^5^ and a minimum, belonging to sample O8N_*f*_H_*m*_, of 91 ± 13 copies per μL of extracted DNA (Supplementary Table 1). Despite substantial differences in median values, likely due to large copy number variation within groups as a function of OysterID, there was no significant difference in 16S rRNA copy number as a function of “first” or “group” (Supplementary Table 2).

**FIGURE 2:**
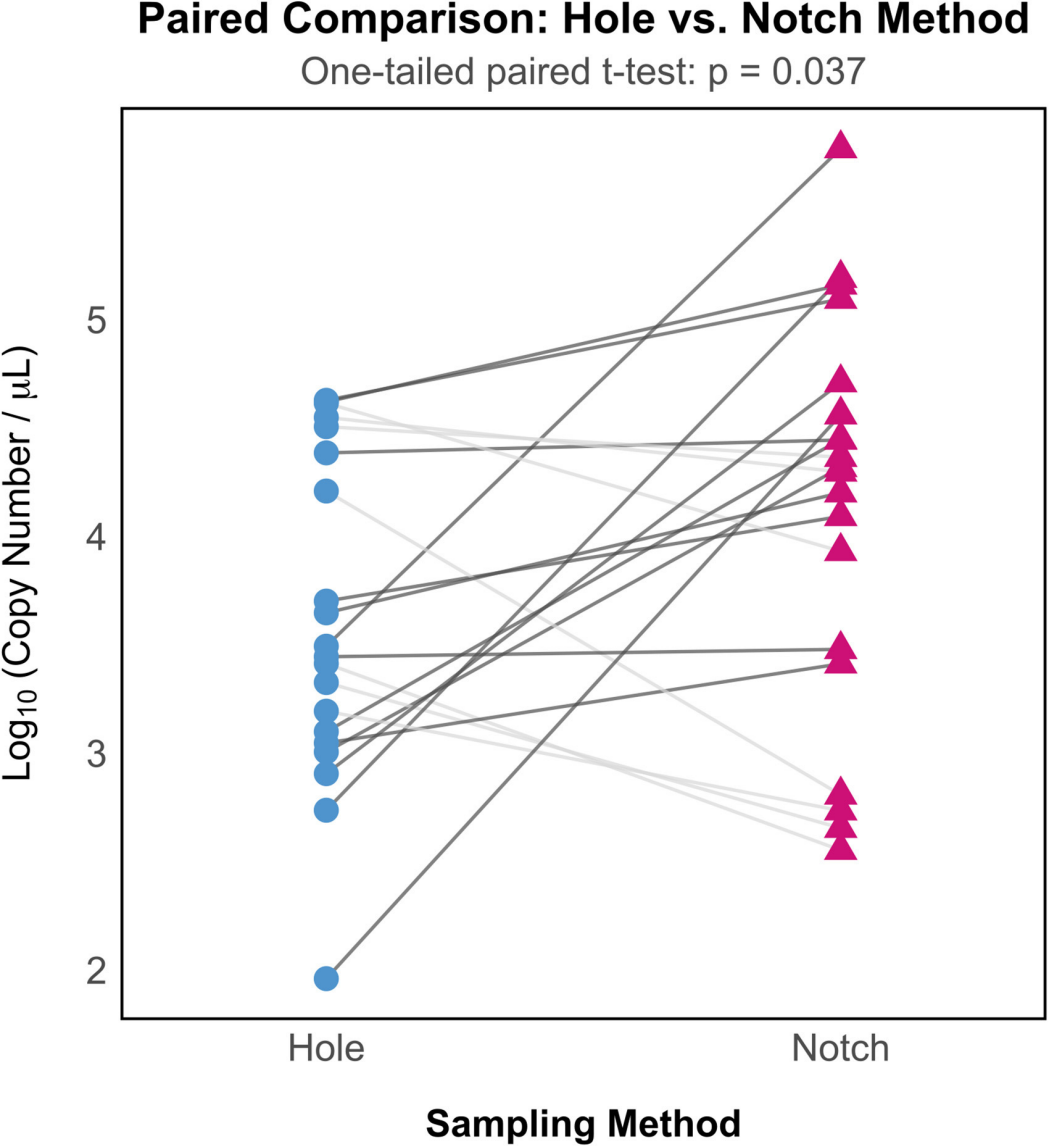
Slopegraph showing the impact of sampling method on *log*_10_-transformed 16S rRNA copy number values: paired samples are connected by OysterID.

To mitigate the impact of interindividual variability in microbial loads, paired samples, referring to N_*m*_ and H_*m*_ samples originating from the same oyster, were evaluated to determine whether the notch sampling method was associated with higher 16S rRNA copy number. A one-tailed Student’s *t*-test using *log*_10_-transformed 16S rRNA copy number values showed a significantly higher mean log-concentration (4.11 ± 0.92) in notch samples as compared to hole samples [3.62 ± 0.76; *t*(19) = 1.90, *p* = 0.037; Figure 2].

### 3.2 16 S rRNA amplicon sequencing

In total, extracted DNA from 106 of the 134 DNA extraction subsamples was successfully sequenced resulting in data for 31 of the 40 original hemolymph samples and at least one replicate of each environmental and DNA extraction control. This creates a distribution of 10 samples in “group” N_*f*_N_*m*_, 6 in N_*f*_H_*m*_, 8 in H_*f*_N_*m*_, and 7 in H_*f*_H_*m*_. The sequencing run yielded an average of 1.0 ± 0.2 × 10^5^ paired-end reads per sample. An average of 87.4 ± 10% of these reads were maintained following merging, quality filtering, and chimera removal (Supplementary Table 3). Two low-read outliers, O11H_*f*_H_*m*_ replicate A and O18H_*f*_H_*m*_ replicate B, having 1,273 and 2,537 raw paired-end reads, respectively, were eliminated from downstream analyses.

### 3.3 Alpha diversity analysis

In terms of total alpha diversity, no statistically significant nor nearly significant differences were observed as a function of any predictor variable (Supplementary Table 4). The variation in both Simpson and Shannon diversity indices driven by predictor variables including “group,” “first,” “method,” and “order” was a small fraction of the variation within these same variables. The two models for Simpson and Shannon diversity using “first” identity as the predictor variable, for example, generated *R*^2^-values of 0.004 and 0.002, respectively, highlighting the limited extent to which these variables drive patterns of alpha diversity. Running these same models with 20 additional alpha diversity metrics showed the same general trend. Overall, this reflects either insufficient variation between the groups or insufficient statistical power to detect any differences.

### 3.4 Beta diversity analysis

**FIGURE 3:**
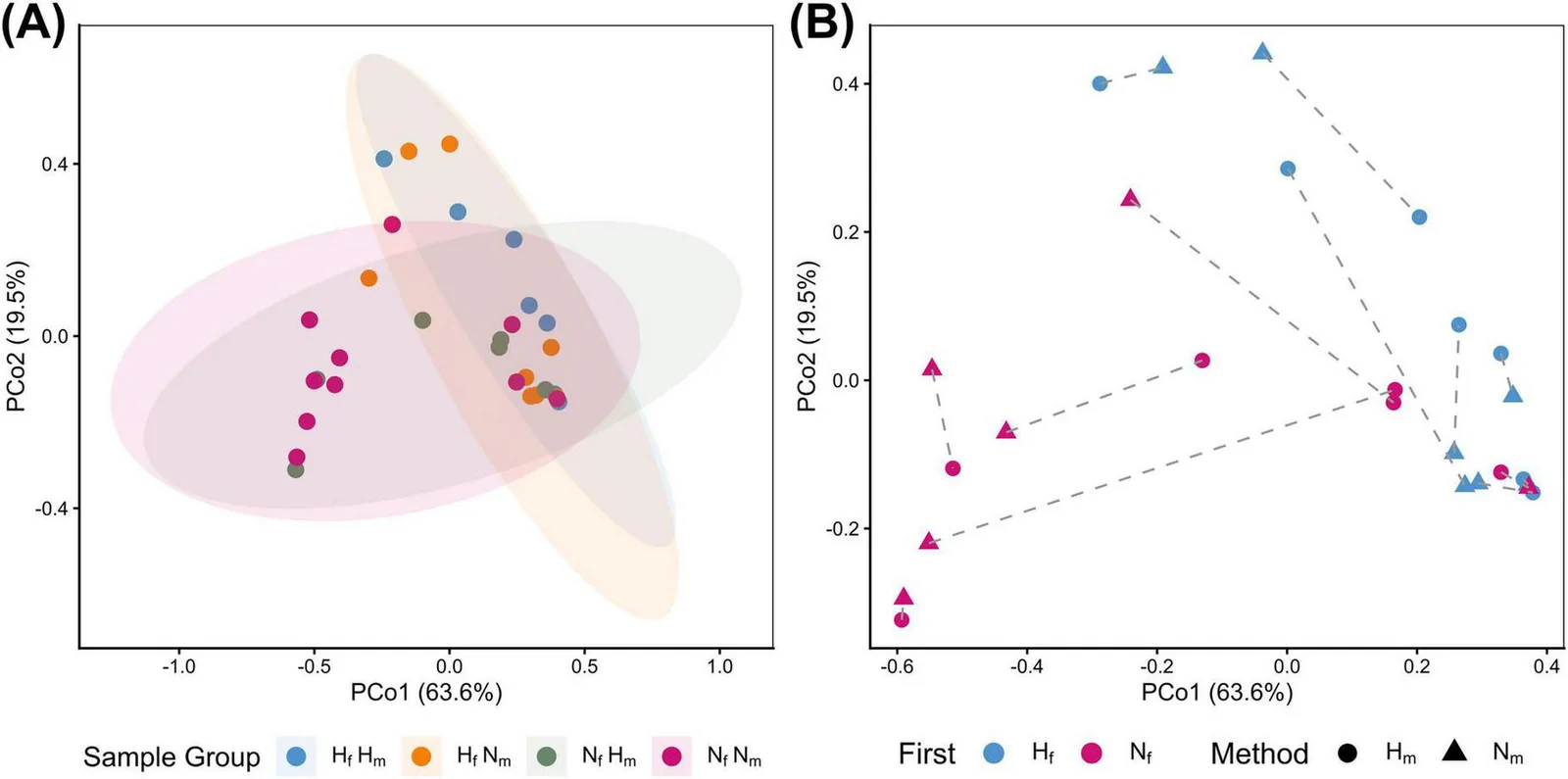
PCoA plots showing the level of similarity between samples. **(A)** Samples clustered by “group” with 95% confidence intervals. **(B)** Samples designated by “first” and “method”; dashed lines indicate pairs of samples linked by OysterID.

Microbial community composition most significantly differed between samples when clustered by the first sampling method used, “first” (Pseudo-F_1,__29_ = 6.86; *R*^2^ = 0.191; p_*adj*_ = 0.006), and by the combined group identity, “group” (Pseudo-F_3,__27_ = 2.51; *R*^2^ = 0.218; *p* = 0.034; Table 3). Comparisons between each unique pair of “group” identities showed no effect with the exception of nearly significant differentiation between N_*f*_N_*m*_ and H_*f*_H_*m*_ samples (Pseudo-F_1,16_ = 5.44; *R*^2^ = 0.266; p_*adj*_ = 0.096), suggesting that differences between these groups are the primary driver behind the overall significant effect of “group.” Finally, comparisons of both N_*f*_N_*m*_ and H_*f*_H_*m*_ samples with all others, a comparison designed to isolate the specific effects of the notch and hole method, revealed significant divergence in community composition associated with these two “group” identities. PERMANOVA analysis identified these significant differences in data structure for N_*f*_N_*m*_ versus all others (Pseudo-F_1,__29_ = 4.77; *R*^2^ = 0.14; p_*adj*_ = 0.016) and H_*f*_H_*m*_ versus all others (Pseudo-F_1,__29_ = 2.94; *R*^2^ = 0.092; p_*adj*_ = 0.045). The permutation tests to evaluate homogeneity of dispersions between the groups defined by “first” (*p* = 0.249) and “group” (*p* = 0.862) indicated no significant differences in dispersion. These quantified differences between groups are visualized via PCoA plots which show sample clustering predominantly tied to “first” though significant within group variation somewhat blurs that observation (Figure 3A). When connected by OysterID, paired samples showed a generally consistent positive shift, particularly within N_*f*_ samples (N_*f*_N_*m*_ and N_*f*_H_*m*_), along Principal Coordinate 1 from N_*m*_ to H_*m*_ samples (Figure 3B).

**TABLE 3:** PERMANOVA results for all comparisons.

Comparison category	Comparison	Degrees of freedom (df)	Sum of squares (SS)	*R* ^2^	F-statistic	*P*-value	p_adj_
Primary hypotheses	“First”	1, 29	1.167	0.191	6.856	0.002	**0.006**
	“Method”	1, 24	0.112	0.021	0.523	0.169	0.169
	“Order”	1, 24	0.119	0.023	0.555	0.119	0.179
	“Group”	3, 27	1.330	0.218	2.508	**0.034**	NA
Pairwise comparisons	N_*f*_N_m_ vs. N_*f*_H_m_	1, 14	0.090	0.033	0.471	0.281	0.337
	N_*f*_N_m_ vs. H_*f*_H_m_	1, 15	0.975	0.266	5.436	0.016	0.096
	N_*f*_N_m_ vs. H_*f*_N_m_	1, 16	0.744	0.209	4.220	0.026	0.078
	N_*f*_H_m_ vs. H_*f*_H_m_	1, 11	0.396	0.169	2.231	0.093	0.186
	N_*f*_H_m_ vs. H_*f*_N_m_	1, 12	0.256	0.109	1.475	0.230	0.345
	H_*f*_H_m_ vs. H_*f*_N_m_	1, 13	0.073	0.034	0.453	0.500	0.500
Planned contrasts	N_*f*_N_m_ vs. all Others	1, 29	0.862	0.141	4.769	0.008	**0.016**
	H_*f*_H_m_ vs. all others	1, 29	0.562	0.092	2.943	0.045	**0.045**

Bold *p* values indicate statistical significance based on a 0.05 threshold.

### 3.5 Compositional abundance analysis

In total, 286 OTUs were observed across the collected hemolymph samples. Two of these OTUs, OTU127 and OTU179 corresponding to *Poseidonibacter lekithochrous* and *Vibrio mediterranei*, respectively, dominate the taxonomic composition of most samples with the exceptions of O9NfNm, O10NfNm, O13HfHm, O19HfHm, O12HfNm, O18HfNm, and O19HfNm (Supplementary Figure 2). Expanding to the family level, a similar pattern of compositional dominance by the *Arcobacteraceae* and *Vibrionaceae* emerges. Outside of these dominant groups, the following eight families make up the remaining “important OTUs”: *Rhodobacteraceae*, *Shewanellaceae*, *Schleiferiaceae*, *Umboniibacteraceae*, *Fusobacteriaceae*, *Flavobacteriaceae*, *Metamycoplasmataceae*, and *Mycobacteriaceae*.

**FIGURE 4:**
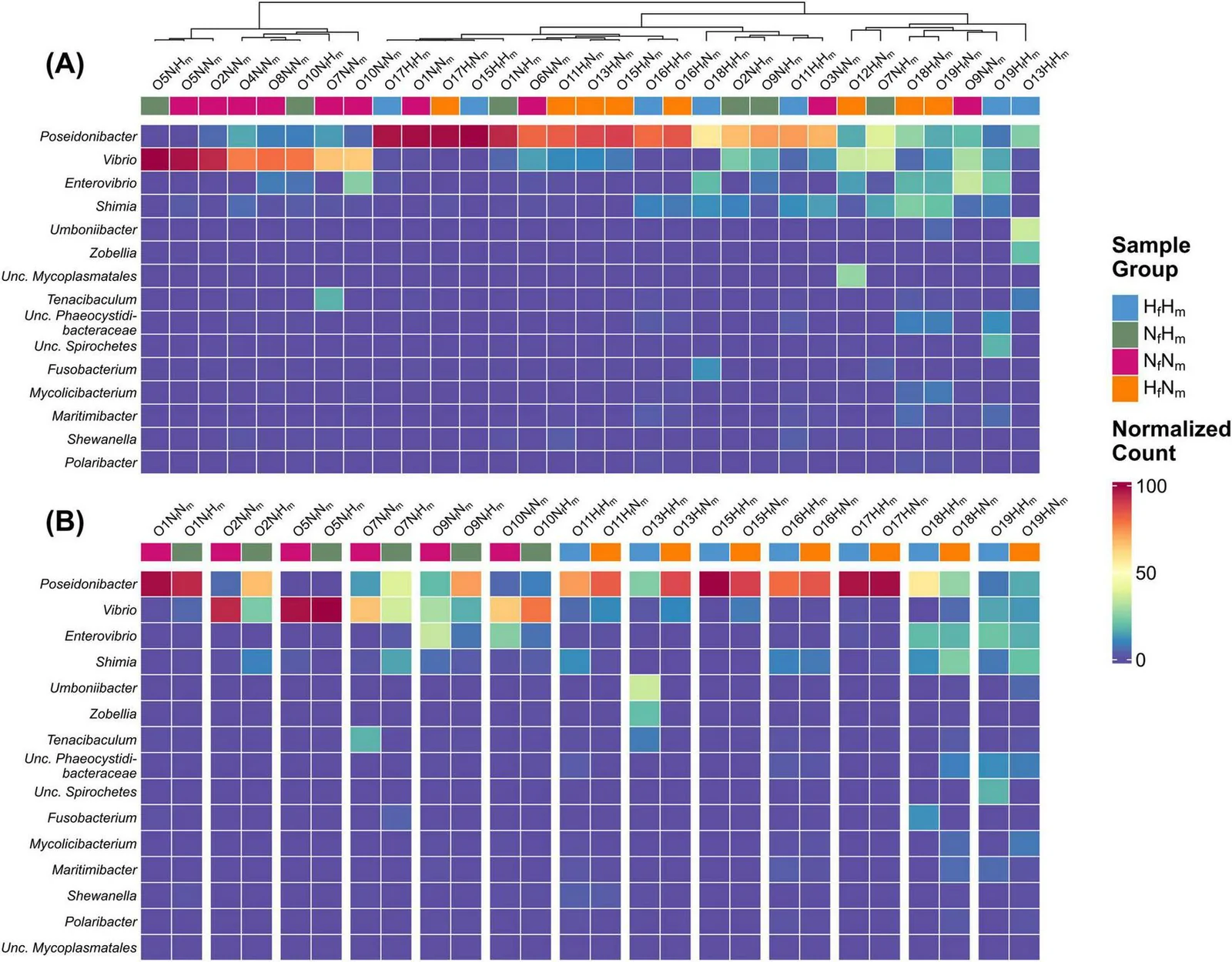
Taxonomic heatmap at the genus level with samples labeled by “group” identity. **(A)** Samples clustered by compositional similarity. **(B)** Samples clustered in pairs by OysterID.

This clear dominance by *Vibrio* and *Poseidonibacter* species, which make up an average of 29 and 50% of reads, is visible in genus level heatmaps (Figure 4A). When paired by OysterID, notch samples appear to show a pattern of increased *Vibrio* and decreased *Poseidonibacter* relative abundance compared to corresponding hole samples (Figure 4B). Samples belonging to the N_*f*_N_*m*_ group in particular appear to be significantly enriched in OTUs belonging to the *Vibrio* genus.

**FIGURE 5:**
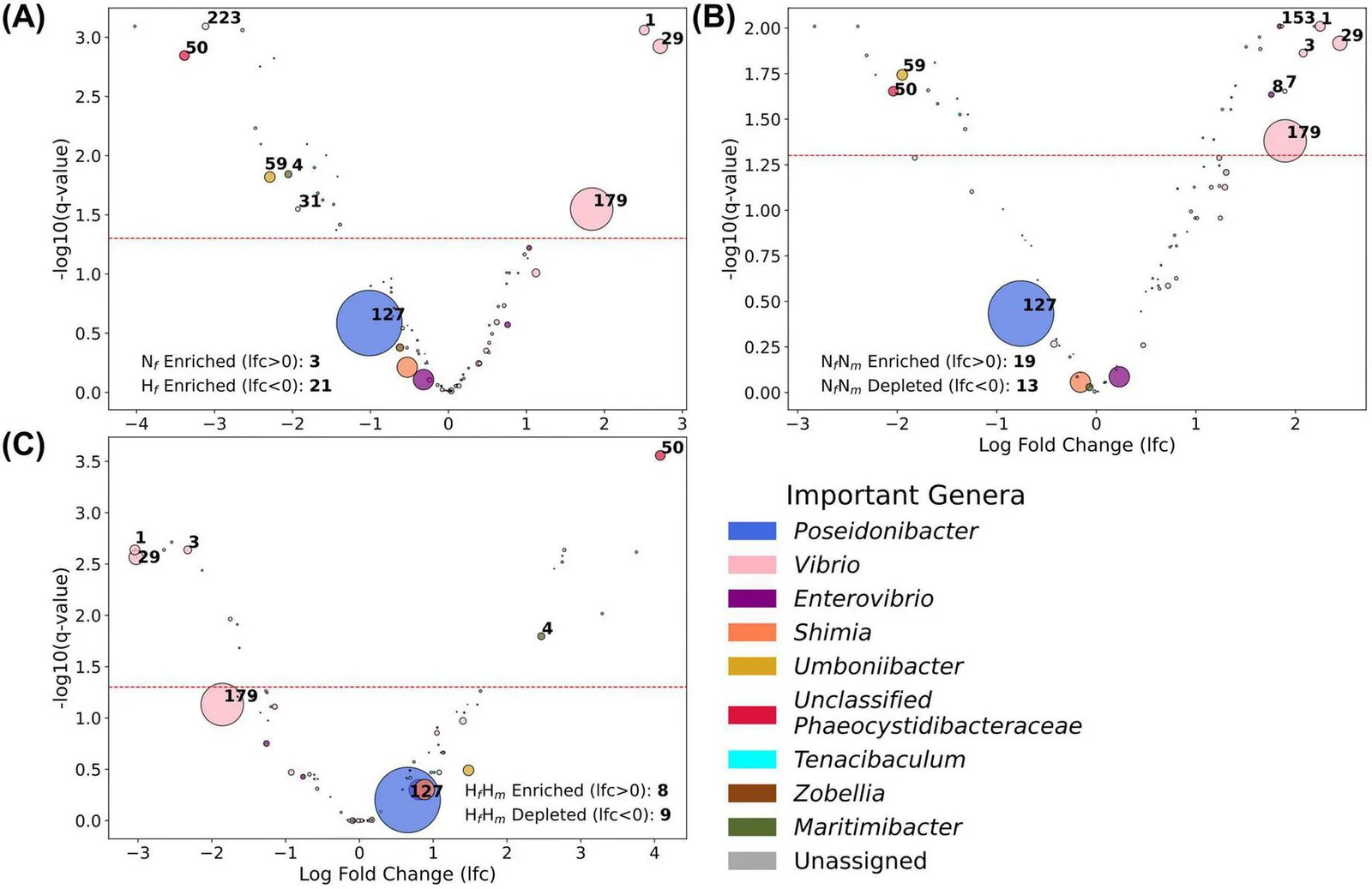
Volcano plots showing differentially abundant OTUs based on **(A)** “first,” **(B)** N_*f*_N_m_ versus all other samples, and **(C)** H_*f*_H_m_ versus all other samples.

As suggested by these observed changes in relative *Vibrio* and *Poseidonibacter* abundances, differences in community composition were driven most strongly by sample grouping, shifting distinctly based on the first sampling method used (“first”), N_*f*_N_*m*_ identity (N_*f*_N_*m*_ versus all other samples), and H_*f*_H_*m*_ identity (H_*f*_H_*m*_ versus all other samples). These prominent baseline patterns were supported by the overall number of differentially abundant OTUs (DA OTUs; defined by an FDR-adjusted *p* < 0.05) across the sample types (Supplementary Table 5). When separated by “first,” a narrow group of three OTUs, all belonging to the *Vibrio* genus, were enriched in N_*f*_ samples, whereas a broader range of 21 OTUs spanning multiple genera were enriched in the H_*f*_ samples (Figure 5A). A generally similar pattern was observed when focusing on N_*f*_N_*m*_ and H_*f*_H_*m*_ “group” identity. A large selection of *Vibrio* was enriched in N_*f*_N_*m*_ samples and depleted in H_*f*_H_*m*_ samples; conversely, a few distinct OTUs, primarily unclassified *Phaeocystidibacteraceae* or those belonging to the *Umboniibacter* genus, were depleted in the N_*f*_N_*m*_ samples and enriched in H_*f*_H_*m*_ samples (Figures 5B,C).

This sampling-driven division along familial lines was further mirrored among the most important community members, with 11 of the 21 pre-identified “important OTUs” (Table 2) being differentially abundant as a function of “first,” N_*f*_N_*m*_ “group” identity, and H_*f*_H_*m*_ “group” identity (Table 4). Specifically, five OTUs belonging to the *Vibrionaceae* family were the exclusive representatives from this prioritized list enriched in N_*f*_N_*m*_ or N_*f*_ samples more broadly. Conversely, six of the OTUs, belonging to the *Rhodobacteraceae*, *Shewanellaceae*, *Schleiferiaceae*, *Flavobacteriaceae*, *Umboniibacteraceae* (Ho et al., 2025), and *Mycobacteriaceae* families, were either enriched in H_*f*_H_*m*_ and H_*f*_ samples or depleted in the N_*f*_N_*m*_ samples. Finally, a notable downward trend in abundance was observed in the notch-sampled cohorts for OTU127 (*Poseidonibacter lekithochrous*), which on average is the single most abundant organism across all samples. Many individual samples within the N_*f*_N_*m*_ group in particular exhibited very low levels of this taxon relative to the study-wide average (Supplementary Table 6), corresponding to a negative log-fold change (LFC) abundance in N_*f*_ samples that did not quite reach a statistically significant degree of depletion.

**TABLE 4:** Degree of differential abundance for important OTUs.

OTU ID	Family	“First” LFC	N_*f*_N_m_ LFC	H_*f*_H_m_ LFC
OTU1	*Vibrionaceae*	N_f_ Enriched (2.51)	Enriched (2.25)	Depleted (−3.04)
OTU3	*Vibrionaceae*	NA	Enriched (2.08)	Depleted (−2.33)
OTU4	*Rhodobacteraceae*	H_f_ Enriched (−2.05)	NA	Enriched (2.46)
OTU29	*Vibrionaceae*	N_f_ Enriched (2.72)	Enriched (2.45)	Depleted (−3.03)
OTU31	*Shewanellaceae*	H_f_ Enriched (−1.93)	NA	NA
OTU50	*Schleiferiaceae*	H_f_ Enriched (−3.39)	Depleted (−2.04)	Enriched (4.08)
OTU59	*Umboniibacteraceae*	H_f_ Enriched (−2.29)	Depleted (−1.95)	NA
OTU130	*Flavobacteriaceae*	H_f_ Enriched (−2.64)	Depleted (−1.32)	Enriched (2.78)
OTU153	*Vibrionaceae*	NA	Enriched (1.84)	NA
OTU179	*Vibrionaceae*	N_f_ Enriched (1.84)	Enriched (1.90)	NA
OTU223	*Mycobacteriaceae*	H_f_ Enriched (−3.12)	NA	NA

The qPCR-scaled absolute abundance of *Vibrio* (calculated as described in Methods section 2.4.3) showed statistically significant differences across the combined “group” identities (N_*f*_N_*m*_, N_*f*_H_*m*_, H_*f*_H_*m*_, H_*f*_N_*m*_), a pattern driven primarily by a clear separation between the N_*f*_N_*m*_ and H_*f*_H_*m*_ groups; mean qPCR-scaled absolute abundance values for *Vibrio* were more than 10-fold higher in the N_*f*_N_*m*_ group as compared to the H_*f*_H_*m*_ group (11,792 vs. 1,016 estimated gene copy numbers). This dynamic was supported by a Kruskal-Wallis rank sum test evaluating “group” identity as the grouping factor and qPCR-scaled absolute *Vibrio* abundance as the response variable [Kruskal-Wallis H(3) = 9.87, *p* = 0.020, ϵ^2^ = 0.33]. A subsequent Dunn’s *post-hoc* pairwise comparison confirmed a significant difference exclusively between the N_*f*_N_*m*_ and H_*f*_H_*m*_ groups (*Z* = −3.03, p_*adj*_ = 0.015), with no other pairs displaying significant variations (p_*adj*_ > 0.05; Table 5). A similar and more pronounced pattern emerged for *Vibrio* relative abundance, which also varied significantly across the groups, shifting 16-fold from 52.6% in the N_*f*_N_*m*_ group to 3.2% in the H_*f*_H_*m*_ group. For relative abundance, pairwise comparisons identified significant shifts between two distinct pairs of groups: N_*f*_N_*m*_-H_*f*_H_*m*_ (*Z* = −3.30, p_*adj*_ = 0.006) and N_*f*_H_*m*_-H_*f*_H_*m*_ (*Z* = −2.78, p_*adj*_ = 0.027), with the overall differences validated by the primary model [Kruskal-Wallis H(3) = 14.93, *p* = 0.002, ϵ^2^ = 0.50; Table 5].

**TABLE 5:** Kruskal-Wallis rank sum test and Dunn’s *post-hoc* pairwise comparisons for *Vibrio.*

Comparison	Degrees of freedom (df)	KW Chi-squared (χ^2^)	*P*-value
“Group”—estimated absolute abundance	3	9.865	**0.020**
“Group”—relative abundance	3	14.931	**0.002**
**Comparison—estimated absolute abundance**	**Z-score**	***P*-value**	**p_adj_**
N_*f*_N_m_ vs. N_*f*_H_m_	−1.499	0.134	0.536
N_*f*_N_m_ vs. H_*f*_H_m_	−3.027	0.002	**0.015**
N_*f*_N_m_ vs. H_*f*_N_m_	−2.047	0.041	0.203
N_*f*_H_m_ vs. H_*f*_H_m_	−1.290	0.197	0.591
N_*f*_H_m_ vs. H_*f*_N_m_	−0.365	0.715	0.715
H_*f*_H_m_ vs. H_*f*_N_m_	−1.006	0.314	0.629
**Comparison—relative abundance**	**Z-score**	***P*-value**	**p_adj_**
N_*f*_N_m_ vs. N_*f*_H_m_	−0.149	0.881	0.881
N_*f*_N_m_ vs. H_*f*_H_m_	−3.298	0.001	**0.006**
N_*f*_N_m_ vs. H_*f*_N_m_	−2.453	0.014	0.057
N_*f*_H_m_ vs. H_*f*_H_m_	−2.783	0.005	**0.027**
N_*f*_H_m_ vs. H_*f*_N_m_	−2.012	0.044	0.133
H_*f*_H_m_ vs. H_*f*_N_m_	−0.892	0.372	0.745

Bold *p* values indicate statistical significance based on a 0.05 threshold.

In contrast to the variations observed in *Vibrio*, *Poseidonibacter* metrics showed no statistically significant differences as a function of “group” for either qPCR-scaled absolute or relative abundance. For instance, while the mean relative abundance of *Poseidonibacter* appeared nearly two-fold lower in the N_*f*_N_*m*_ group compared to the H_*f*_H_*m*_ group (34.5% vs. 62.7%, respectively), their average qPCR-scaled absolute values were essentially identical (15,692 vs. 16,058 estimated gene copy numbers). Kruskal-Wallis tests confirmed this lack of group-driven variation for both estimated counts [Kruskal-Wallis *H*(3) = 3.18, *p* = 0.365] and relative abundance [Kruskal-Wallis *H*(3) = 5.60, *p* = 0.133; Supplementary Table 7]. Due to this absence of overall structural variance, no *post-hoc* pairwise comparisons were performed for *Poseidonibacter* in either case.

## 4 Discussion

Accurate characterization of oyster hemolymph-associated bacterial communities depends not only on downstream sequencing and analysis, but also on how hemolymph is physically accessed and processed. In this study, comparison of notch- and hole-collected samples provides a framework for evaluating whether commonly used hemolymph sampling approaches introduce method-associated differences in recovered bacterial profiles.

A critical component of the experimental design, necessary to evaluate the impact of sampling method within the same oyster, was repeated hemolymph sampling with alternating methods. Such an approach, however, has the potential to be limiting in that the second sample can be impacted by both the previously used sampling method directly and by the fact that the animal necessarily has less hemolymph and an altered structure of the adductor muscle. By altering the order that the methods were used, hence splitting the oysters into notch-first (N_*f*_) and hole-first (H_*f*_) categories, the impact of both sampling method order and repeated sampling can be quantitatively evaluated. The lack of any significant effect of “order” demonstrates that the impact of these factors was minor relative to the impact of sampling method which will be discussed throughout the rest of this section.

With this methodological basis established, the higher 16S rRNA copy number values in notch compared to hole samples, regardless of sampling order, appears to support the hypothesis that usage of the notch sampling method introduces a specific and directional bias favoring higher bacterial loads—increased bacterial DNA that possibly, rather likely, represents contamination from non-hemolymph tissues. Moreover, these heightened bacterial DNA concentrations associated with samples collected using the notch method reflect a disproportionate increase in *Vibrio* spp. (Table 5; discussed below). These results, in combination with those discussed in the remainder of this section, highlight that bacterial communities derived from invasive tissue sampling methods reflect patterns uniquely characteristic to the chosen sampling method; patterns that must be accounted for and mitigated when analyzing community composition.

Overall, the general compositional pattern of observed hemolymph bacterial communities matches that previously observed in oysters with strong representation by *Gammaproteobacteria*, *Alphaproteobacteria*, *Bacteroidia*, and *Campylobacterota* (formerly *Epsilonproteobacteria*) (Lokmer and Mathias Wegner, 2015; Pimentel et al., 2021; Dupont et al., 2020). At the order and genus level the bacterial communities observed here strongly match those observed in eastern oysters reared in Maryland (Gignoux-Wolfsohn et al., 2024). Insofar as taxonomic composition is concerned, the most prominent pattern present throughout the samples was the differing ratios of *Vibrio* to *Poseidonibacter*. While the former group is widely recognized as a major component of the oyster hemolymph microbiota (Dupont et al., 2020; Lokmer et al., 2016; Wendling et al., 2014), for a variety of reasons including many recent changes in taxonomic classification, the latter genus is less universally observed. Though *Arcobacter*, the genus in which *P. lekithochrous* existed prior to its reclassification in 2018 (Pérez-Cataluña et al., 2018), has been reported in various marine organisms including oysters, its presence is far less consistent than other genera including *Vibrio* (Lokmer and Mathias Wegner, 2015; Pimentel et al., 2021; Dupont et al., 2020; Romero et al., 2002). Thus, the overwhelming abundance of *P. lekithochrous*, making up over 50% of the reads for 16 out of 31 hemolymph samples, stands out compared to existing literature.

The lack of statistically significant differences in alpha diversity, despite observed differences in bacterial community composition and abundance of many genera, is likely driven by two main factors. For one, the relatively small number of samples within each group, with sequencing data being obtained for 31 of the original 40 hemolymph samples, reduces statistical power when applying various model types. This effect is particularly pronounced for paired comparisons, such as “method” and “order,” as the loss of nine samples more drastically reduces the number of full pairs that can be used in the models. Amplifying this is the presence of many structural zeroes throughout the dataset, further complicating calculations of alpha diversity that are tied to the presence and distribution of rare taxa. The second factor may be the observed compositional dominance of two OTUs, *Poseidonibacter lekithochrous* and *Vibrio mediterranei*, which together make up on average over 70% of reads across all samples. The overwhelming presence of these OTUs across samples masks the impacts of numerous rare taxa on alpha diversity but has less of an effect on beta diversity, which captures change in overall composition between samples rather than the level of diversity within them.

While high *P. lekithochrous* was common across samples, the broader community composition, and thus ratio of *Poseidonibacter* versus other genera, primarily *Vibrio*, differed strongly as a function of “group” and “first.” This raises the question of why these differences are observed when samples are clustered by “first” and not by “method.” However, the fact that this is not observed, rather than indicating a true lack of effect, may result from the previously discussed diminished statistical power when doing paired comparisons. Unfortunately, comparisons based on “method” must utilize a pairwise model design as it compares samples within the same OysterID, meaning within-oyster variation must be controlled for relative to between-oyster variation.

This phenomenon is further complicated by the nature of the “first” variable. In addition to samples from the same oyster not being independent due to this shared origin, they are also not independent because the first method used to collect hemolymph from a given oyster has the potential to influence the microbial community of the second hemolymph sample from that oyster. When a notch sample is taken from an oyster, we hypothesize that such an action has the potential to introduce contaminant bacteria from trace pallial fluid, mucous membranes, or other non-hemolymph tissue. If this were the case, this contamination would persist when taking the follow-up hemolymph sample using the hole method. Similarly, there is the potential for the first usage of the hole method to somehow disturb the hemolymph in such a way that the second sample, taken using the notch method, is influenced. Thus, despite categorizing them as separate predictor variables, “first,” particularly within the context of comparing N_*f*_N_*m*_ and H_*f*_H_*m*_ samples, is not isolated from the effects of the sampling method but is, in part, reflective of it. Given this, significant differences between samples based on “first,” with respect to both PERMANOVA results and DA analysis, are influenced by the effect of sampling method. It is worth noting that the taxonomic heatmap (Figure 4B) and PCoA plot (Figure 3B) with samples linked by OysterID, appear to demonstrate within-oyster shifts in community composition that cannot be explained by “order” and thus are most likely associated with sampling method.

This conclusion, that sampling method itself contributes to differences in sample microbial community composition, is supported by the degree of differentiation seen in the composition of N_*f*_N_*m*_ versus H_*f*_H_*m*_ samples based on both taxonomic heatmaps (Figure 4A and Supplementary Figure 3) and PERMANOVA analysis (Table 3). Comparing these two groups is essentially the same as comparing the notch method being used on one oyster to the hole method being used on a different oyster; a reasonable experimental design given sufficient sample size and shared holding conditions. Since the oysters employed for this study possessed the same genetic background, were maintained in the same tank, and had samples collected in a staggered manner to reduce batch effect, differences between the groups are most consistent with an effect of the two sampling methods. As such, the significant effect of “group” identity on sample community composition being primarily driven by differences between N_*f*_N_*m*_ and H_*f*_H_*m*_ samples emphasizes that sampling method is a statistically detectable contributor to the composition of hemolymph bacterial communities.

**FIGURE 6:**
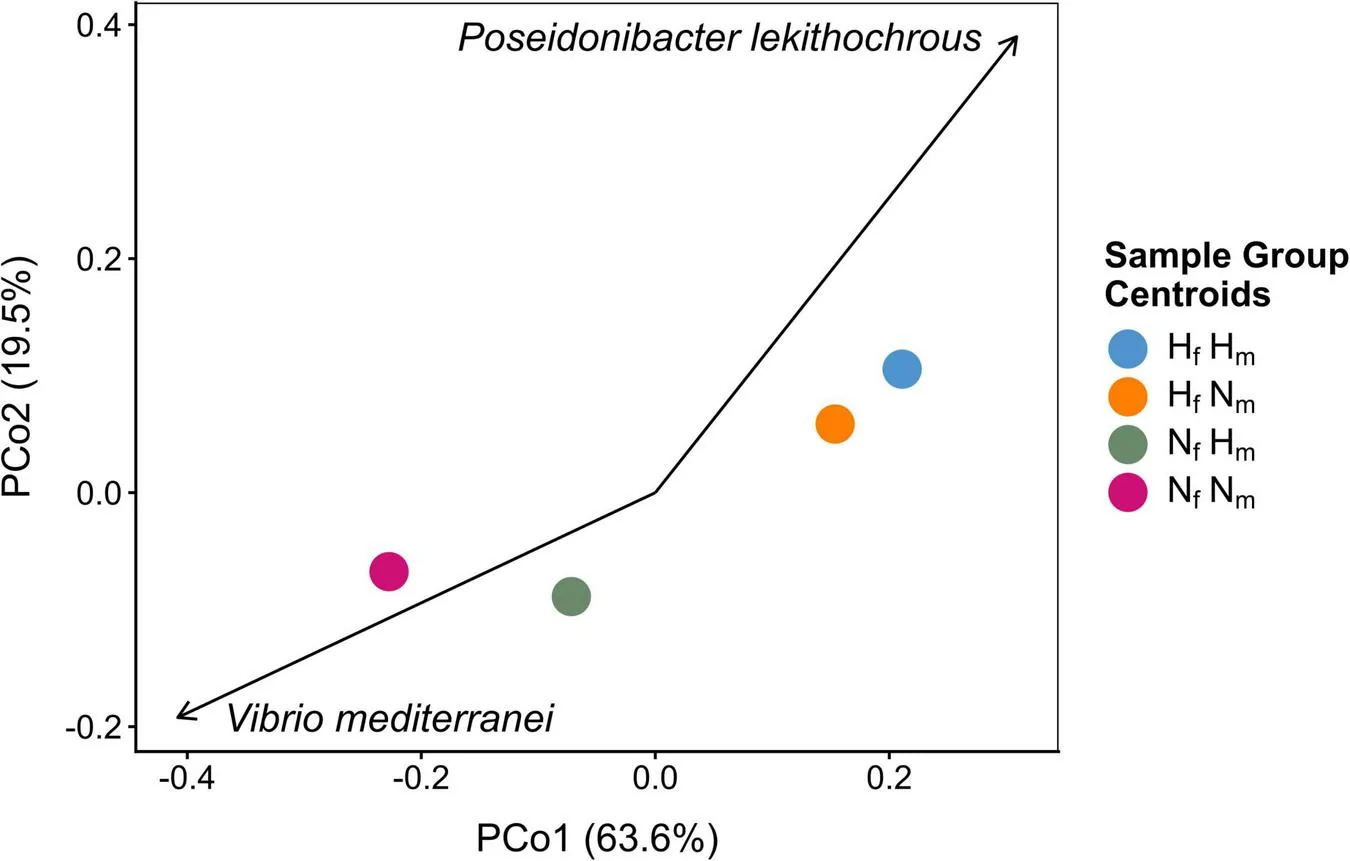
Annotated PCoA plot showing the relationship of *P. lekithochrous* and *V. mediterranei* relative abundance with overall differences in bacterial community composition.

Supported by differential abundance analysis (Figure 5), the significant enrichment of *Vibrio* OTUs in N_*f*_N_*m*_ samples and their near-absence in H_*f*_H_*m*_ samples (Figure 4) suggests that their abundance in hemolymph, along with the abundance of *Vibrio* in hemolymph more broadly, is likely reflective of a notch method-specific sampling artifact. The effect of this bias can be seen in the annotated PCoA plot (Figure 6). Differences in the relative abundance of *V. mediterranei* and *P. lekithochrous*, the two most abundant bacterial species within our samples with the former being significantly enriched in N_f_ samples, play the strongest role relative to all other OTUs in driving the locations of sample groups on the PCoA plot and thus the composition of samples within these groups.

The implications of this finding are significant as these very same *Vibrio* are frequently reported as potentially pathogenic members of the oyster’s normal resident hemolymph microbiota, with many such findings of *Vibrio* presence in hemolymph using the notch method or not specifying their hemolymph collection method (Wendling et al., 2014; Wegner et al., 2025; Lokmer et al., 2016; Dupont et al., 2020; Desriac et al., 2014; Zhang et al., 2018; Faury et al., 2004; Gay et al., 2004; Li et al., 2022). While the results of this study do not challenge the existence of these *Vibrio* and certainly not the role they play in driving oyster mortality and dysbiosis, they do call into question our understanding of the dynamics and spatial distribution of these bacteria in oyster tissues in general and hemolymph in particular, and the extent to which past research on this topic has been influenced by sampling method bias.

Based on the DA analysis results, and more specifically the depleted levels of certain non-*Vibrio* OTUs in notch-associated samples, one may initially think that N_*f*_N_m_ samples have lower numbers of OTUs such as *P. lekithochrous*. However, comparing the estimated absolute read counts, rather than the relative abundance values used for *ancombc2*-based DA analysis, reveals that this is not the case. While the mean relative abundance of *Poseidonibacter* drops by half in the N_*f*_N_m_ group relative to H_*f*_H_m_, the average absolute counts remain unchanged. The illusion of *Poseidonibacter* depletion in notch samples is a mathematical artifact resulting from their signal being swamped by the massive absolute influx of *Vibrio* in the N_*f*_N_m_ samples. Ultimately, these results imply that while the notch sampling method did collect a broadly similar bacterial community as that captured using the hole method, it introduced a profound, method-specific influx of *Vibrio* on top of the baseline hemolymph microflora.

These striking differences underscore two important and related points. The first is that relative abundances on their own have the potential to be significantly misleading when evaluating bacterial community composition (Maghini et al., 2024; Props et al., 2017). When comparing bacterial communities across tissues, between treatment groups, or, in this case, between sampling types it can be easy to assume that overall bacterial loads across samples are similar meaning that relative abundances should reflect absolute abundances. However, without specific validation of this assumption using absolute counts, such assumptions can lead to highly misleading interpretations of data. In this case the addition of a large number of *Vibrio* reads in N_*f*_N_m_ samples creates a substantially reduced relative abundance of various other genera potentially leading one to assume that the samples have uniformly different bacterial community structures rather than a significant absolute enrichment of one dominant genus potentially associated with sampling-method bias.

The second point is tied to this extreme and disproportionate level of *Vibrio* itself. More than just confusing our understanding of *Vibrio* dynamics in hemolymph, a tissue highly relevant to immune function, such inflated levels of *Vibrio* can largely swamp out the signal of other significant bacteria such as *P. lekithochrous* generating misleading conclusions regarding the composition of hemolymph bacterial communities and thus the impact that these bacteria can have on oyster health and disease. *Poseidonibacter lekithochrous* for instance is a widely distributed bacteria across a range of marine species including the great scallop, various corals, and the Chilean mussel (Diéguez et al., 2017; Girija et al., 2024; Montúfar-Romero et al., 2025; Kim et al., 2025). This broad marine distribution, combined with some evidence suggesting that *P. lekithochrous* may be a hemolymph-specific symbiont (Lokmer and Mathias Wegner, 2015), means that the notch method may be masking the presence of core microbial community members in N_*f*_N_m_ samples.

## 5 Conclusion

This study provides valuable methodological insight into not only oyster hemolymph collection, but also the broader importance of optimizing microbiome sampling while accounting for and clearly communicating potential biases. By collecting hemolymph using both the notch and hole method in the eastern oyster, we were able to characterize and compare the bacterial communities in the resulting samples. Significant differences in broad patterns of community composition, highlighted by the differences between the N_*f*_N_m_ and H_*f*_H_m_ groups, are consistent with the effects of sampling method: an impact that is visible between paired samples within oysters. A number of key community members, most notably members of the *Vibrio* genus and *Poseidonibacter lekithochrous*, were identified via differential abundance analysis and their respective PERMANOVA *R*^2^ values as strong drivers of the observed differentiation.

The substantial and selective enrichment of *Vibrio*, particularly *V. mediterranei*, in notch-associated samples indicates that this method has the potential to introduce contamination from non-hemolymph tissues. This pattern suggests that alternative approaches, such as the hole method, warrant further consideration when designing oyster microbiome experiments. Further research employing biological validation controls and stronger mechanistic evaluations is necessary to confirm if and how the notch sampling method introduces contamination from non-hemolymph tissues (e.g., mucosal epithelia). The research described here should prompt further critical investigations of tissue sampling methods within the context of microbiome research. Regardless of method used, our findings prompt a call to action for microbiome researchers across biological systems. Given the potential impact of sampling method on tissue-derived microbial communities, researchers should not only carefully consider their sampling method choice, but also recognize and account for the biases inherent to it.

## Data Availability

The datasets generated for this study can be found in the NCBI Sequence Read Archive (SRA) under accession number of SRP677661.
